# Phages on filaments: A genetic screen elucidates the complex interactions between *Salmonella enterica* flagellin and bacteriophage Chi

**DOI:** 10.1371/journal.ppat.1011537

**Published:** 2023-08-03

**Authors:** Nathaniel C. Esteves, Danielle N. Bigham, Birgit E. Scharf

**Affiliations:** Dept. of Biological Sciences, Virginia Polytechnic Institute and State University, Blacksburg, Virginia, United States of America; University of Toronto, CANADA

## Abstract

The bacterial flagellum is a rotary motor organelle and important virulence factor that propels motile pathogenic bacteria, such as *Salmonella enterica*, through their surroundings. Bacteriophages, or phages, are viruses that solely infect bacteria. As such, phages have myriad applications in the healthcare field, including phage therapy against antibiotic-resistant bacterial pathogens. Bacteriophage χ (Chi) is a flagellum-dependent (flagellotropic) bacteriophage, which begins its infection cycle by attaching its long tail fiber to the *S*. *enterica* flagellar filament as its primary receptor. The interactions between phage and flagellum are poorly understood, as are the reasons that χ only kills certain *Salmonella* serotypes while others entirely evade phage infection. In this study, we used molecular cloning, targeted mutagenesis, heterologous flagellin expression, and phage-host interaction assays to determine which domains within the flagellar filament protein flagellin mediate this complex interaction. We identified the antigenic N- and C-terminal D2 domains as essential for phage χ binding, with the hypervariable central D3 domain playing a less crucial role. Here, we report that the primary structure of the *Salmonella* flagellin D2 domains is the major determinant of χ adhesion. The phage susceptibility of a strain is directly tied to these domains. We additionally uncovered important information about flagellar function. The central and most variable domain, D3, is not required for motility in *S*. Typhimurium 14028s, as it can be deleted or its sequence composition can be significantly altered with minimal impacts on motility. Further knowledge about the complex interactions between flagellotropic phage χ and its primary bacterial receptor may allow genetic engineering of its host range for use as targeted antimicrobial therapy against motile pathogens of the χ-host genera *Salmonella*, *Escherichia*, or *Serratia*.

## Author summary

Multidrug-resistant (MDR) bacteria kill over one million people per year worldwide. For instance, MDR typhoid fever is a significant concern in developing countries. As we precipitously approach a post-antibiotic society, bacteriophage therapy is a promising solution to the antibiotic crisis. Bacteriophage χ is a virus capable of infecting three species of pathogenic bacteria via their flagella, a significant virulence factor. Flagellum-dependent phages exploit an evolutionary tradeoff: a bacterium suppressing motility to avoid infection by such a phage would likely attenuate its own virulence. This makes flagellum-dependent phages of particular interest for potent therapy against MDR pathogens. In this study, we unveil crucial information about the mechanism of infection of *Salmonella enterica* by χ, knowledge which has the potential to lead to genetic modification of χ phage’s host range and subsequent clinical applications. Similar studies can be conducted in other phage systems to broaden humanity’s repertoire of potent therapeutic bacteriophages.

## Introduction

*Salmonella enterica* is a very broad species of Proteobacteria responsible for millions of bacterial infections per year worldwide [1]. With greater than 2,600 serotypes contained within six subspecies [2], *S*. *enterica* is an extremely diverse bacterial species. Serotypes are broadly categorized as typhoidal or non-typhoidal. Typhoidal *Salmonellae* cause the potentially deadly disease typhoid fever, which leads to numerous deaths particularly in developing countries [3]. Non-typhoidal Salmonellosis (NTS) is a generally self-limiting bacterial gastroenteritis [4], which presents with diarrhea, nausea, and vomiting and carries a slight risk of death from dehydration or invasive disease, with certain serotypes being more likely than others to be invasive [5]. NTS is the leading cause of bacterial foodborne illness worldwide, resulting in an estimated 93 million cases per year and hundreds of thousands of deaths [1,4]. Multidrug resistance (MDR) is a growing concern in *S*. *enterica* [1,3,6]. MDR typhoid fever is a particularly serious problem, as people with untreated typhoid infections have a high rate of mortality [6,7]. A potential solution to treat infections caused by increased bacterial antibiotic resistance is bacteriophage therapy [8–10].

Bacteriophages, or phages, are viruses which solely infect bacteria without attacking eukaryotic cells [11]. Their host specificity, efficiency of killing, and self-replicating nature make phages remarkable candidates for potent antimicrobial therapy [12–14]. In fact, phages have been used for this purpose beginning over a century ago [15], but were largely abandoned with the invention and popularization of antibiotics [12]. As we move toward a post-antibiotic era, phage therapy has seen a significant resurgence as a valid medical treatment [13,16]. The host specificity of phages greatly improves their usefulness as therapeutics, as a phage treatment can be specifically tailored to a particular pathogenic bacterium, avoiding disruption of the delicate balance of natural bacterial flora. Phage cocktails, mixtures of phages, can be used when broader spectra of activity are needed [17]. More information is required about phage infection mechanisms for the true potential of phage therapy to be realized.

All phages begin their infection cycle by binding to a bacterial-encoded receptor [18,19]. This receptor is recognized and bound to via a phage-encoded receptor binding protein (RBP) [18]. Phage receptor diversity is extremely broad, with various phages being capable of utilizing lipopolysaccharide, pili, porins, or other cell membrane components as receptors [19,20]. One unique phage infection mechanism is that of the flagellotropic (flagellum-dependent) phages. This diverse category of viruses shares the common feature of using the bacterial flagellum as its primary receptor [20].

The bacterial flagellum is a rotary appendage that propels bacteria through their surroundings via swimming motility [21–23] or on surfaces via swarming motility [24]. The flagellum is composed of three major subsections: basal body, hook, and filament [25]. The basal body is a complex of over twenty proteins, which form the motor responsible for torque generation and the secretion system machinery required for export of flagellar proteins [25]. The rod of the motor is attached to the flexible hook [26] which is, in turn, attached to the helical filament [21]. The filament is a long, rigid propeller that translates motor rotation into forward propulsion. It is a polymer composed of a protein called flagellin, often designated FliC in organisms such as *Escherichia coli* [27] and *S*. *enterica* [28]. Up to 30,000 individual flagellin monomers polymerize into a helix with eleven-fold radial symmetry to form a single filament [29]. Flagella and flagellins are potent virulence factors in many pathogenic bacteria for a multitude of reasons [30–33]: (i) motility itself gives bacteria a competitive advantage [34]; (ii) flagella play roles in cell invasion, biofilm formation, and adhesion to surfaces [1,31,34]; and (iii) flagellin is a potent antigen recognized by toll-like receptor 5 in humans [35,36].

Flagellin proteins are extremely variable between bacterial strains, both by amino acid sequence and length [27,37]. For instance, the hypervariable D3 domains of flagellin share merely 15.5% amino acid sequence identity between the closely related *S*. *enterica* serotypes Typhimurium and Enteritidis (Table 1). Certain bacteria, such as the *Yersiniaceae* species *Serratia marcescens*, have highly truncated FliC proteins [38]. In many enteric bacteria, flagellin is composed of seven domains: D0, D1, and D2 at the N- and C-termini, and a single, central D3 [39,40]. In the three-dimensional FliC structure, each N-terminal domain is located adjacent to its C-terminal counterpart. NTD2, CTD2, and D3 form the antigenic region, facing outward in the assembled flagellar filament [40]. These antigenic domains are hypervariable with D3 being the most variable and typically having very little conservation between different serotypes [37]. The D2 and D3 domains also greatly vary in length from over 1,000 amino acid residues to essentially lacking these antigenic domains [20,37]. For instance, *Caulobacter crescentus* encodes six different short flagellins, all of which lack D2 and D3 domains [41]. Variation in the flagellin antigenic domains likely plays a role in pathogenesis [27]. In contrast, structural domains NTD0, NTD1, CTD1, and CTD0 are highly conserved even across different phyla of bacteria and are crucial for filament core formation and filament assembly [37,40,41].

**Table 1:** Susceptibility phenotypes of a subset of *Salmonella enterica* serotypes and their flagellin (H) antigens [2]. The symbol (-) indicates no phase 2 flagellin is produced by that serotype. None of the serotypes listed produce phase 3 flagellin. A comprehensive list of all tested serotypes and their H antigen formulae is found in S1 Table.

Serotype	Phase 1 H antigen	Phase 2 H antigen	χ host phenotype
Abortusovis	c	1,6	Resistant
Choleraesuis	c	1,5	Susceptible
Enteritidis	g,m	-	Resistant
Heidelberg	r	1,2	Resistant
Javiana	l,z_28_	1,5	Susceptible
Montevideo	g,m,s	-	Resistant
Schwarzengrund	d	1,7	Susceptible
Typhimurium	i	1,2	Susceptible

In addition to the primary structure, there are other significant differences between the flagellins of various Enterobacterales bacteria. Most *S*. *enterica* serotypes undergo flagellin phase variation between two flagellins via a unique transcriptional regulatory mechanism [42]. The *fljAB* operon is under the control of an inversible promoter, which switches direction mediated by the Hin invertase. When the promoter faces in the antisense direction relative to the *flj* operon, *fljA* and *fljB* are not transcribed. When the promoter faces forwards, *fljA* and *fljB* are transcribed. The gene product FljB is phase 2 flagellin, while FljA is a transcriptional repressor of *fliC*. This ensures that only one flagellin gene is expressed at any given time [42]. The inversion is a rare event [42,43], and under normal circumstances *Salmonella* cells will only produce filaments composed of a single flagellin type at a time. More rarely, some *S*. *enterica* serotypes produce a third flagellin, generally given the designation FlpA. This third flagellin gene is typically plasmid-borne and can therefore be unstable, may not be consistently present in all strains of a particular serotype, and can be horizontally transferred to other serotypes [44]. *E*. *coli* and *S*. *marcescens* each typically have only a single flagellin. *E*. *coli* and *S*. *marcescens* also lack the flagellin lysine-N-methyltransferase FliB present in *S*. *enterica* [45,46]. This enzyme adds methyl groups to lysine residues primarily in the antigenic domains NTD2, CTD2, and D3 of both FliC and FljB [47]. Flagellin methylation in *Salmonella* has implications for adhesion, immune evasion, and virulence [47]. Similarly to *E*. *coli* and *S*. *marcescens*, *S*. *enterica* has never been shown to post-translationally modify its flagellins in other ways such as glycosylation [48].

Bacteriophage χ (Chi) is a flagellum-dependent (flagellotropic) phage infecting multiple species of enteric bacteria [49–51]. The infection process of χ is hypothesized to follow a nut-and-bolt mechanism, which postulates that the phage attaches to the filament within the grooves formed by the flagellin monomers and is brought down to the cell surface by the rotation of the flagellum, much like a nut moving down a bolt [20,52,53]. The requirements for infection by χ are thus very specific. Cells that lack flagella or that have paralyzed flagella are unable to be infected by χ [49]. Control of motor rotation through the chemotaxis system has also been shown to be important for χ infection, as cells with flagella that only rotate clockwise are resistant to χ [52]. Phage χ is known to infect *S*. *enterica* [20,50,54,55], *E*. *coli* [20,49], and *S*. *marcescens* [51], all of which are capable of pathogenesis [1,56,57]. The breadth of the Enterobacterales and relative lack of χ phage research means that other bacterial species may be susceptible as well. The status of flagella and motility as potent virulence factors makes flagellotropic phages of particular interest as powerful antimicrobial agents against clinically significant bacteria [20]. Phages targeting virulence factors or antibiotic resistance mechanisms impose an exploitable evolutionary tradeoff: a bacterium which downregulates expression of a virulence factor such as flagellin to avoid infection by a phage would likely attenuate its own virulence [20,58]. Bacteriophage χ has an unusual host range in that it infects bacteria across two families, the *Enterobacteriaceae* and *Yersiniaceae*, but infects only certain serotypes within *S*. *enterica* subsp. *enterica*. Subtle differences between *Salmonella* serotypes therefore must determine whether a strain is susceptible or resistant to χ phage. Very little has been uncovered about the factors contributing to this host range, but flagellar structure is a likely candidate. This is because the flagellum is the primary receptor for χ [49,53], and flagellin antigen is a key differentiating factor between *Salmonella* serotypes [2]. We have recently identified a potential secondary cell surface receptor for χ. The multidrug efflux pump AcrABZ-TolC is essential for χ infection as its deletion results in complete abolishment of infection without halting motility [54]. The essential nature of an antibiotic resistance mechanism for infection further increases the attractiveness of χ for clinical use, as avoidance of phage infection by mutation of AcrABZ-TolC is an additional evolutionary tradeoff that can possibly be exploited by coadministration of phage and antibiotic [58,59]. In this study, we used targeted mutagenesis and heterologous expression of mutant flagellin to determine which structural domains of the flagellar filament are essential for χ infection, broadening knowledge on a phage with potential clinical applications, in addition to employing motility assays with FliC domain mutants to further understand the function of *Salmonella* flagella.

## Results

### Susceptibility phenotypes cannot be determined solely from antigenic formulae

We tested numerous *S*. *enterica* serotypes with representative Phase 1 and Phase 2 antigens for χ susceptibility to determine whether a correlation could be found between antigen type and phage susceptibility. We employed qualitative phage spot assays to determine χ susceptibility. A small subset of tested strains indicated that bacteriophage χ is only capable of infecting certain *S*. *enterica* serotypes (Table 1). More comprehensively, our qualitative serotype susceptibility testing (total n = 37) demonstrated that approximately half of the tested serotypes (S1 Table) are hosts for χ (n = 19), while the other half is resistant (n = 18). There are monophasic, biphasic, and triphasic serotypes in both the resistant and susceptible category, ruling out a requirement for multiple flagellins for χ infection. We have noted that strains with the g flagellin antigen, including serotype Enteritidis, are always resistant to χ, in agreement with observations by Meynell [50]. Other than that, no clear correlation between serotype and susceptibility was found. It is worth noting that the host range of χ is significantly different from the host range of the very closely related phage YSD1 [60]. Additionally, ser. Typhimurium FliC expressed in ser. Enteritidis does not confer a host phenotype (S1 Table).

### Motility is significantly reduced when *fliC* is expressed from pBS1316

**Fig 1:**
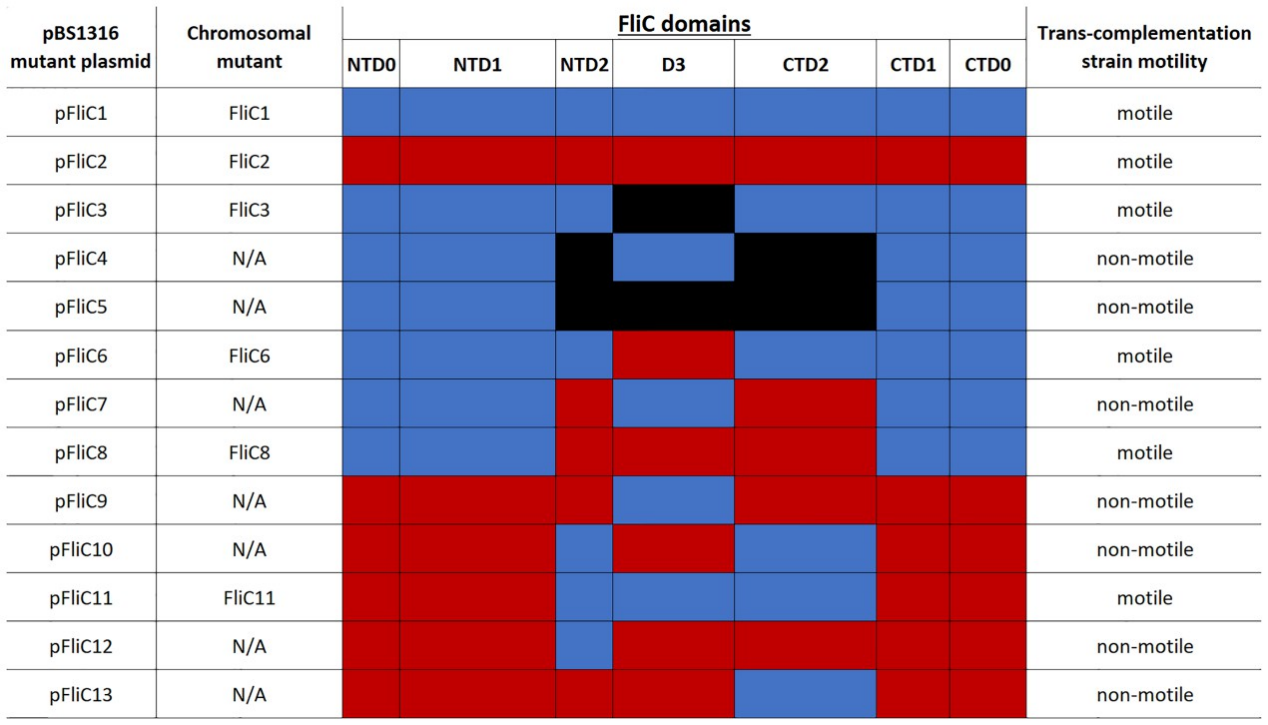
Abbreviated names of FliC domain mutants. Both plasmids and their corresponding chromosomal mutants are shown, where applicable. N/A indicates that a corresponding chromosomal mutant was not constructed, as the trans-complementation strain was not motile. The identity of the domains of flagellin in each mutant is color coded, with blue indicating domains from ser. Typhimurium strain 14028s, red indicating domains from ser. Enteritidis strain P125109, and black indicating a deletion of that entire domain. The trans-complementation strain motility phenotypes given in the rightmost column dictated whether a chromosomal mutant was constructed for that particular strain.

**Fig 2:**
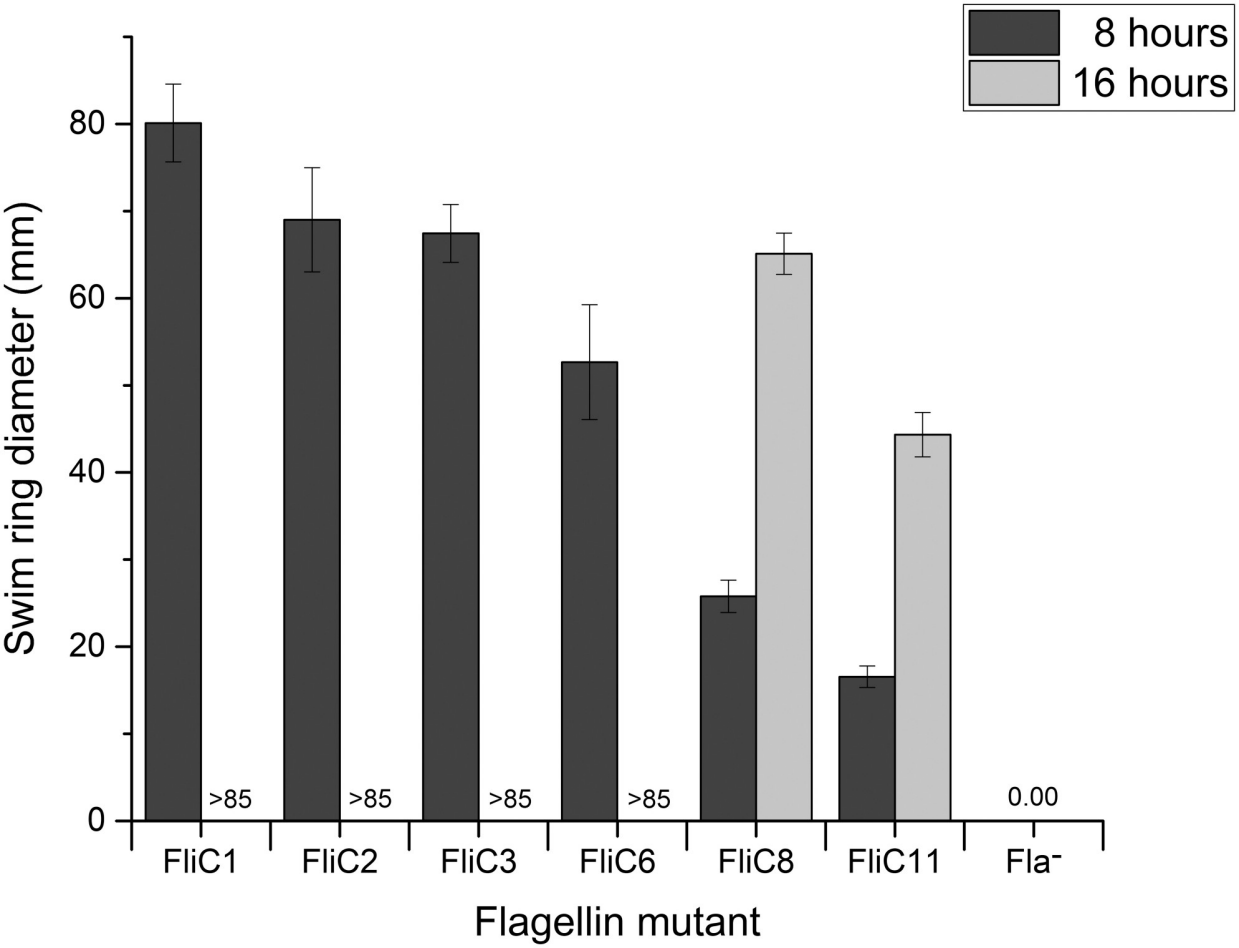
Swimming motility of flagellin mutants quantified on 0.3% agar MSB swim plates. Values are given as swim ring diameter in millimeters. Swim rings were measured at 8 hours and 16 hours post-inoculation. Each mutant’s swim ring data are representative of three technical replicates for each of three biological replicates. Swim rings for strains FliC1, FliC2, FliC3, and FliC6 reached the edge of their respective plates (85 mm) prior to the second time point and were therefore not measured at 16 hours and are marked as >85. Negative control Fla^-^ is marked with 0.00, as this strain is non-flagellated and formed no swim ring after the full 16 hours of incubation. All mutants’ motility defects are statistically significant compared to FliC1, calculated using Student’s T-Test.

We constructed numerous flagellin mutants and chimeras (Fig 1) by combining domains from the host serotype Typhimurium and the non-host serotype Enteritidis, as well as introducing deletions of entire domains. These included mutations of the antigenic domains NTD2, D3, and CTD2, as well as the structural NTD0, NTD1, CTD1, and CTD0 domains. FliC sequences are aligned in S1 Fig, and the residue assignments are listed in Table 2. Mutant flagellins were first expressed from a novel flagellin expression vector, which is based on plasmid pTrc99a. This vector was constructed to include the entire ser. Typhimurium *fliC* promoter in addition to known regulatory elements in the 5’ UTR [28,63]. Despite the use of this vector, motility was significantly reduced with trans-complemented flagellin compared to flagellin expressed from the chromosome. We also noted a greater level of variability in motility. For this reason, we only report qualitative motility data for the trans-complementation mutants, which are given in the rightmost column of Fig 1, and performed subsequent, quantitative analyses with chromosomal *fliC* mutant strains. Quantification of motility was analyzed on soft agar swim plates, and the data are given in Fig 2. We found that plasmids pFliC4, pFliC5, pFliC7, pFliC9, pFliC10, pFliC12, and pFliC13 did not confer motility to ser. Typhimurium Fla^-^. Conversely, plasmids pFliC1, pFliC2, pFliC3, pFliC6, pFliC8, and pFliC11 rescued motility in ser. Typhimurium Fla^-^ to varying degrees on swim plates or when viewed using phase contrast microscopy. Thus, we constructed chromosomal mutants for the latter group. The non-host ser. Enteritidis only possesses FliC, and it is unknown whether the FliB methylase of ser. Typhimurium would methylate ser. Enteritidis flagellin. Therefore, flagellin mutants were constructed in a Δ*fljBA* Δ*fliB* background to eliminate the variables of flagellin phase variation and flagellin methylation [46,48], and we refer to this strain as WT or FliC1 in the following sections.

**Table 2:** Domain definition of *S. enterica* serotypes Typhimurium and Enteritidis FliC used for fliC mutant construction according to ser. Typhimurium literature and sequence homology [40]. There is no full consensus on the domain definition in all bacterial species, thus values may vary slightly between literature sources. Amino acid sequence identities and similarities between domains of ser. Typhimurium 14028s FliC and ser. Enteritidis P125109 FliC are given. Identities and similarities were calculated by EMBOSS Needleman-Wunsch algorithm (EMBL-EBI) [61,62].

Domain	ser. Typhimurium AAs in 14028s	ser. Enteritidis AAs in P125109	Identity	Similarity
NTD0	1–44	1–44	93.2%	97.7%
NTD1	45–177	45–177	80.5%	88.0%
NTD2	178–190	178–190	16.7%	16.7%
D3	191–290	191–300	15.5%	20.6%
CTD2	291–406	301–416	23.4%	35.9%
CTD1	407–453	417–463	74.5%	87.2%
CTD0	454–495	464–505	95.2%	100.0%
Full length	1–495	1–505	52.4%	61.8%

### *Salmonella* ser. Typhimurium cells expressing ser. Enteritidis *fliC* are motile and χ resistant

**Fig 3:**
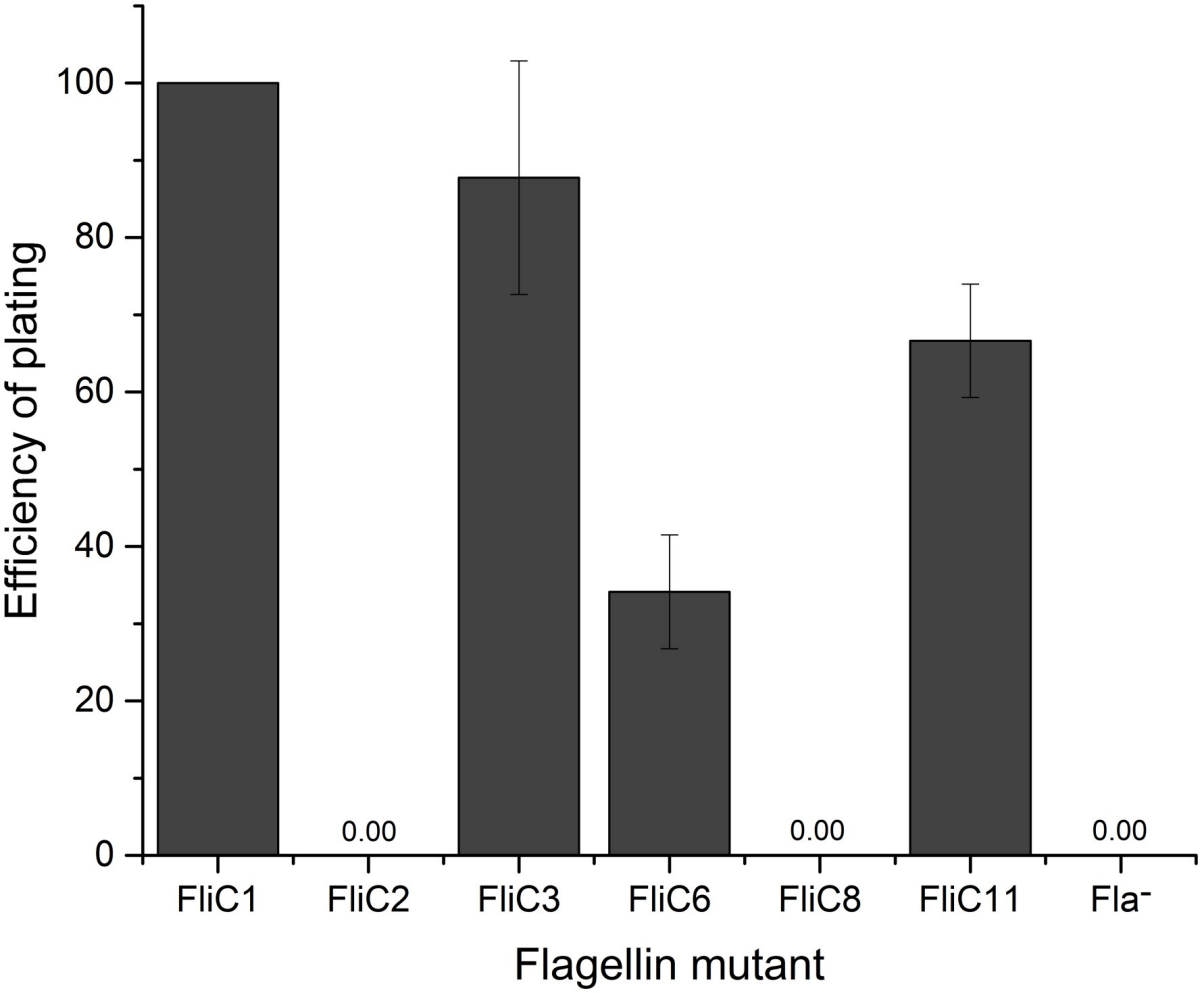
Efficiency of plating (EOP) data for FliC mutants. All EOP values are given as percentage of wild type (FliC1) and consist of three replicates. Error bars are given as standard deviation. Mutants which exhibited zero plaque formation are denoted with 0.00. The reduction in EOP seen with mutant FliC3 is not statistically significant when compared to FliC1. The EOP values of FliC6 and FliC11 are statistically significant from FliC1 and from each other (P<0.05), calculated via Student’s T-Test.

**Fig 4:**
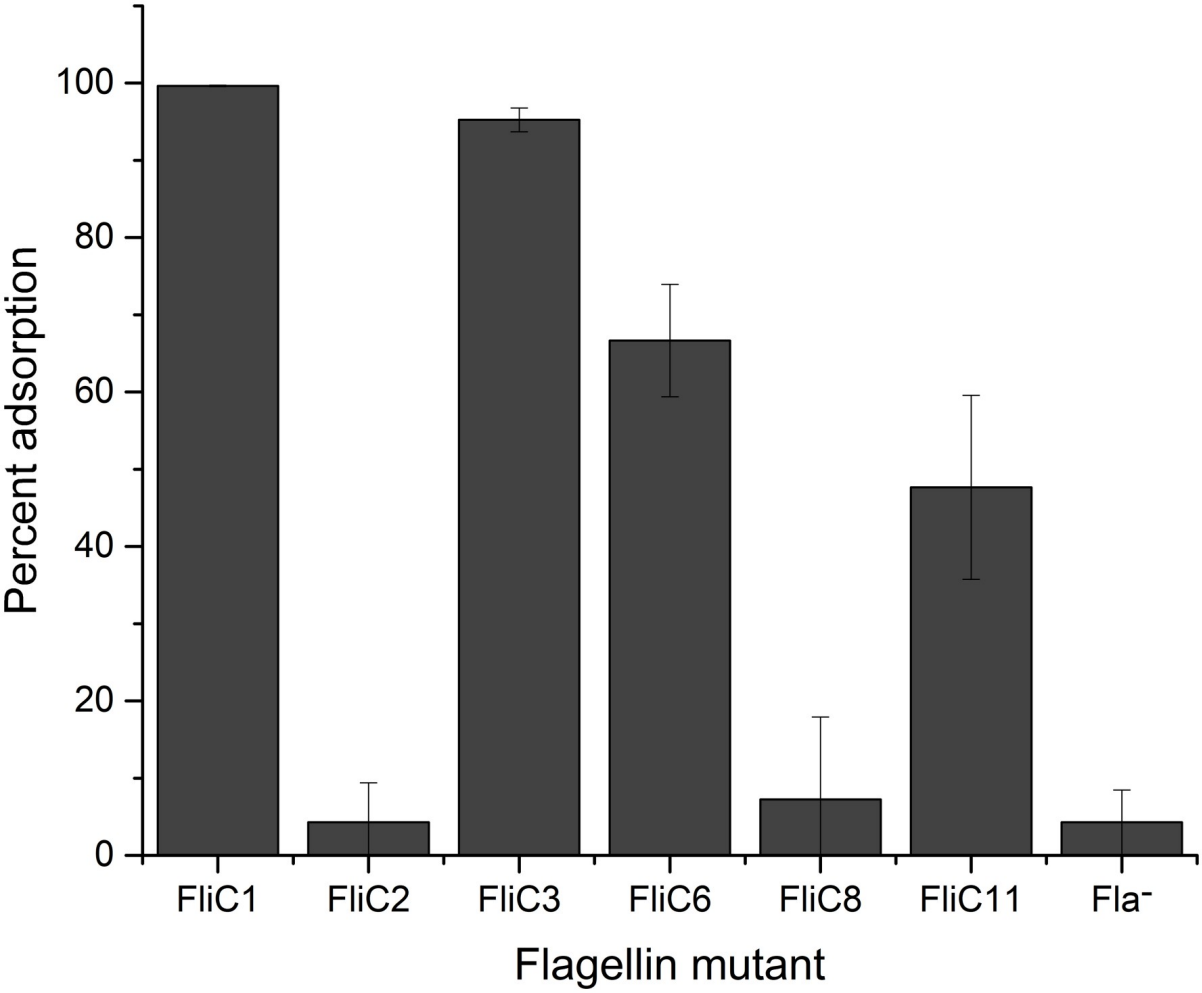
Percentage of phage particles adsorbed to cells after an incubation time of ten minutes. All adsorption values are significantly reduced as compared to FliC1 (P<0.05), calculated using Student’s T-Test. Adsorption values for FliC2 and FliC8 are not significantly different compared to the Fla^-^ negative control. The difference between FliC6 and FliC11 is not statistically significant. All values consist of three replicates and error bars are provided denoting standard deviations.

Strains with chromosomal *fliC* mutations all exhibited increased motility as compared to their plasmid-borne counterparts (Fig 2). This allowed the collection of quantitative EOP and adsorption data. EOP values were calculated as percentage of FliC1 (wild type ser. Typhimurium FliC without expressing FliB or FljB) (Fig 3), and adsorption is given as percentage of phage particles adsorbed after a ten-minute incubation (99% for FliC1) (Fig 4). More importantly, FliC2 (wild type ser. Enteritidis FliC expressed in ser. Typhimurium) exhibits only slightly reduced (86% of FliC1 motility; Fig 2) compared to cells producing native ser. Typhimurium FliC. Despite its high motility, FliC2 was χ resistant, without any plaque formation (EOP = 0) (Fig 3). The EOP assay is highly sensitive and capable of detecting an EOP approximately 10^8^-fold lower than FliC1. As FliC2 did not allow phage production or even adsorption, flagellar filaments composed of a non-host FliC entirely prevent infection of and binding to an otherwise χ susceptible strain (Fig 4). From this we conclude that production of a flagellar filament composed of FliC from a non-host serotype is sufficient to fully block χ infection in a host serotype, signifying that flagellin structure is a key factor determining host range.

### D3 alone has only a small impact on motility and phage interactions

After determining that a non-host flagellin gene expressed in a host strain prevents χ binding and infection, we sought to investigate the potential role of FliC D3 as this is the most variable domain. This goal was accomplished by constructing D3 deletion and substitution mutants. Despite the loss of 100 amino acid residues, a strain with a deletion of the central antigenic ser. Typhimurium FliC D3 (FliC3) was only slightly reduced in its motility (84% of FliC1; Fig 2). A similar phenotype was seen when ser. Typhimurium D3 was replaced with D3 from ser. Enteritidis FliC (FliC6), although this strain displayed a more significant reduction in swim ring diameter with 66% of FliC1. More interestingly, both FliC3 and FliC6 remained susceptible to χ phage, with FliC3 exhibiting an EOP statistically indistinguishable from FliC1 and FliC6 being susceptible to a lesser degree (EOP = 34%). FliC3 presented only a modest reduction in phage adsorption (95%), while FliC6 showed a more pronounced reduction to 67%, indicating a reduced quality of binding in the presence of a non-host D3. Since some mutant strains with large motility defects were still χ susceptible, it rules out the possibility that the modest motility defect of FliC2 described above is causing χ resistance. These data lead to the conclusion that while D3 is not required for binding, a non-host D3 can weaken the quality of binding.

### D2 domains are indispensable for motility and determine χ susceptibility

After determining that D3 is dispensable in χ susceptibility, we next investigated the role of the D2 domains. Deletion and substitution mutants were constructed, with mutations in individual D2 domains, both D2 domains, or D2 and D3 domains combined. The N- and C-terminal D2 antigenic domains are in close proximity in assembled flagellar filaments [40]. Interactions between the D2 domains are known to contribute to the overall stability of the filament. *S*. *enterica* flagellar filaments composed of mutant flagellin monomers lacking portions of the outer domains have been shown to be less stable [64]. Additionally, D2 domains transiently interact with each other and with other flagellin domains to overall stabilize the filament [65]. As such, we observed complete abolishment of motility when D2 domains alone were deleted or substituted, for example with complementation plasmids pFliC4, 12, and 13 (Fig 1). Consequently, these strains were χ resistant. On the contrary, when both D2 domains and the D3 domain of ser. Typhimurium were exchanged with their ser. Enteritidis FliC counterparts (FliC8), motility was partially rescued (32% of FliC1). Despite its significant motility, FliC8 was fully resistant to χ (EOP = 0%) and also prevented χ adsorption, as its adsorption value of 7% was not statistically significant from that of the χ resistant strains FliC2 or Δ*fliC*. Moreover, the inverse holds true: when both D2 domains and the D3 domains from ser. Enteritidis FliC were replaced by their ser. Typhimurium equivalents (FliC11), χ susceptibility was restored with an EOP of 67% of FliC1. Interestingly, this mutant’s EOP was significantly higher than the 34% EOP value of FliC6, which possesses the D0, D1, and D2 domains from the susceptible strain. In contrast, the adsorption value of FliC11 (48%), was lower than that of FliC6 (67%), although this difference was not statistically significant (P = 0.09). Although the motility of FliC11 (21% of FliC1) was significantly reduced, it was sufficient to result in χ susceptibility. From these data, taken together with the comparatively minor effects of D3 mutations, we conclude that the D2 domains play a key role in determining χ susceptibility, as substitution of these domains in conjunction with D3 was sufficient to fully abolish binding and infection while retaining motility.

### All mutants produce their respective mutant flagellins

**Fig 5:**
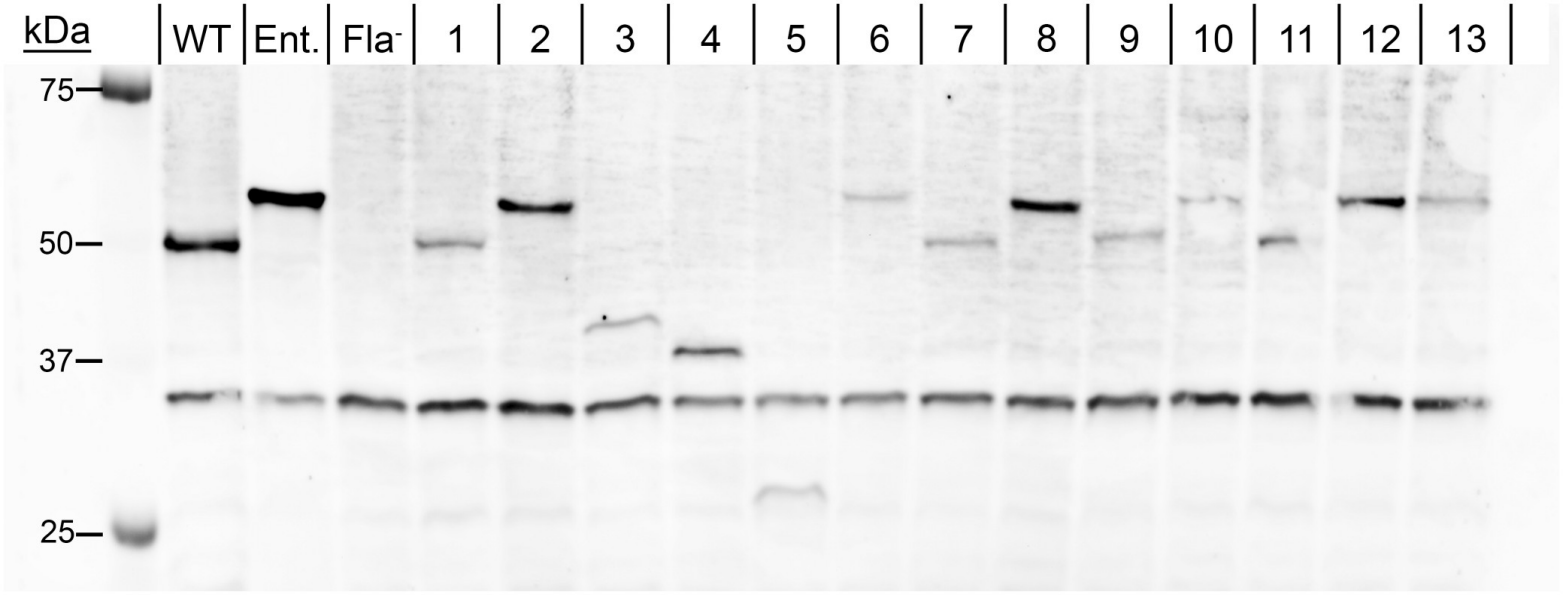
Immunoblot of whole cell lysates from pFliC mutants grown to an OD_600_ of 2.0. Flagellin is detected by BD Difco *Salmonella* anti-H a-z antiserum. Ser. Typhimurium 14028s FliC_ON_ Δ*fliB* (chromosomal mutant FliC1) is denoted as “WT.” Wild type ser. Enteritidis P125109 is denoted as “Ent.” Ser. Typhimurium 14028s Δ*fliC*Δ*fljBA* is denoted as “Fla^-^.” Numbers 1 through 13 refer to the corresponding pFliC plasmid mutants, as described in Fig 1.

After determining mutant motility phenotypes, we analyzed and correlated flagellin production with these phenotypes. Via SDS-PAGE of whole cell lysates, and subsequent Coomassie staining and immunoblotting, we were able to provide evidence that all strains expressed stable flagellins, including those expressing chimeric flagellins (Fig 5 and S2 Fig). Additionally, the commercially available *Salmonella* anti-H a-z antibody detected all flagellin proteins, including those with reduced antigenic domains. Serotype Typhimurium strain 14028s FliC_ON_ Δ*fliB* (chromosomal mutant FliC1; WT) produced an intense flagellin band with an apparent molecular weight of 50 kDa, close to its calculated molecular weight of 51 kDa. The molecular weight of ser. Enteritidis strain P125109 (Ent.) flagellin is only 2 kDa higher than that of 14028s, however, the corresponding FliC band had an apparent molecular weight of 60 kDa. The 51-kDa band of the pFliC1 mutant appeared less intense than WT, likely corresponding to reduced production of WT flagellin. The pFliC2 mutant produced a band of the same molecular weight compared to Ent. The pFliC3 strain (ΔD3) showed a band at approximately 40 kDa, which is similar to the calculated molecular weight of 42 kDa. The non-motile pFliC4 and pFliC5 mutant strains each produced a band at approximately 37 kDa and 28 kDa, respectively, which is in agreement with the calculated sizes of D2, and D2 and D3 deletion mutant flagellins. A faint band could be identified for the motile pFliC6 strain, exhibiting the same apparent molecular weight as Ent. FliC. The faint band in the pFliC7 strain exhibited the expected FliC size (51 kDa), although this mutant was non-motile. The motile pFliC8 strain (D2s and D3s replaced with ser. Enteritidis FliC) produced a band with an anomalous apparent molecular weight similar to the one observed for Ent. FliC. The band in the non-motile pFliC9 strain exhibited an increased intensity with a comparable size to WT FliC. The non-motile pFliC10 strain showed a band at the typical ser. Enteritidis FliC molecular weight. A distinct band was identified for the motile pFliC11 mutant strain, migrating at the typical molecular weight of ser. Typhimurium flagellin. Finally, the non-motile pFliC12 and pFliC13 mutant strains, encoding chimeric FliCs with ser. Enteritidis D3, both produced faint bands with the anomalous apparent molecular weight of ser. Enteritidis FliC. Lastly, the *Salmonella* anti-H a-z antibody recognized a 34 kDa band of similar intensity in all sixteen strains, including the non-flagellated Fla^-^. We identified bands for all mutants that corresponded to FliC or FliC variants. The Coomassie-stained SDS-PAGE gel of whole cell lysates supported our immunoblot findings, although not all flagellin bands were easily discernable from other cellular proteins (S2 Fig). Interestingly, all non-motile strains expressing chimera or domain-deletion flagellin produced variant FliC protein, which excludes the lack of FliC production as the reason for their motility phenotype.

## Discussion

### Strain selection and elimination of phase variation and methylation

In this study, all experiments are conducted in a *fliC*-locked Δ*fliB* ser. Typhimurium 14028s strain. This strain only produces flagellin FliC due to a complete deletion of phase 1 flagellin repressor gene *fljA* and phase 2 flagellin gene *fljB* [42]. Deletion of the *fliB* gene ensures that flagellin is not methylated [46]. Despite methylation not being crucial for χ phage infection, we believed this to be an unnecessary variable, as methylation may alter the quality of phage binding. It is also unknown whether mutant flagellin proteins would be methylated by FliB. We determined that ser. Typhimurium cells expressing only *fliC* or only *fljB* are susceptible to χ (S1 Table). The *fliC* promoter is a classical promoter, while the *fljAB* promoter is complex, as it undergoes Hin-mediated inversion for flagellin phase variation [42]. For this reason, the experiments in this study were carried out with strains expressing only FliC. Serotype Enteritidis was chosen as a non-host strain for multiple reasons: (i) ser. Enteritidis is a common serotype found in cases of gastroenteritis [66,67]; (ii) it is fully resistant to χ; (iii) it only produces a single flagellin without undergoing phase variation; and (iv) its H antigen type is g,m, with g antigen flagellins having been shown to be χ resistant by Meynell [50] and confirmed by our data (S1 Table). It is worth noting that despite being fully resistant to χ, this serotype allows a very small level of adsorption similar to the Fla^-^ strain (Fig 4). We hypothesize that this is likely due to χ being able to bind directly to the flagellar hook at extremely high multiplicities of infection. Phage χ has been demonstrated to bind to polyhook mutants [52], which would suggest that it likely can bind to hooks with wild-type length at high concentrations.

### Trans-complementation of flagellin yields poor motility

We have found that motility is reduced when flagellin is expressed in trans, even when WT *fliC* is expressed from its native ser. Typhimurium P_*fliC*_ promoter. Bacterial flagellar synthesis, including transcription, export, and assembly, is a complex process with multiple regulatory steps and mechanisms [21,25,68]. Regulation of flagellin transcription may not function properly during *in trans* flagellin expression. The 5’ untranslated region (UTR) of *fliC* has been reported to contain regulatory elements in *S*. Typhimurium [28]. In addition, a stem-loop structure that forms between RNA sequences in the *fliC* 5’ UTR and sequences early in the coding region affects translation [69]. Our pBS1316 vector includes these known regulatory elements, but there could be additional, undiscovered regulatory elements located outside of the cloned region. An abridged RNA length in the 3’ UTR could also possibly reduce mRNA stability, as this has been shown to be a phenomenon in various bacteria [70]. Plasmid copy number may also impact expression levels or disrupt the temporal regulation of flagellin expression [71,72]. High copy number may also result in depletion of the flagellar sigma factor, σ^28^, and subsequently impact expression of late flagellum and chemotaxis genes. Additionally, heterologously expressed flagellin may not interact optimally with chaperone proteins, impacting folding and thus function. Regardless of the cause, this reduction in motility was significant enough to make quantitative phage-host interaction data unreliable, an issue that was ameliorated by the generation of chromosomal flagellin mutants.

### The involvement of domains D2 and D3 in motility and phage infection

We showed in this study that deletion of both D2 domains with or without the D3 domain abolishes motility (Fig 1). In addition, these domains cannot be heterologously substituted without the corresponding homologous D3 domain. We hypothesize that interactions between residues within NTD2, D3, and CTD2 mediate folding and assembly of monomers into the flagellar filament. During evolution, these domain interactions developed exclusively within a specific *S*. *enterica* serotype.

Thus, domains from different serotypes may not interact to support filament assembly, as the antigenic domains are known to play a role in filament stability [64]. However, if both NTD2 and CTD2 are substituted along with D3, they can interact with each other to form a functional filament. These constraints in the experimental approach prohibited us from determining whether NTD2, CTD2, or both are involved in χ infection.

Our experiments allow the conclusion that the central D3 antigenic domain by itself is not essential to determine χ phage susceptibility, despite being the most variable domain between *Salmonella* serotypes, as its deletion only mildly affects χ adsorption and does not disturb infection. Thus, D3 is not required for χ phage interaction. This lack of specificity offers a fitness advantage to χ, as subtle differences in the most variable FliC domain will not impact its capacity to infect a large variety of serotypes. We have also found that loss of D3 only has a minor impact on motility in *S*. Typhimurium. From these results, we infer that D3 is not essential for ser. Typhimurium 14028s filament formation or function, which confirms related studies in *E*. *coli* and *S*. Typhimurium LT2 [73,74]. Due to the vast diversity of flagellin alleles in the *S*. *enterica* genus, we cannot exclude the possibility that the D3 domain is essential for motility in certain serotypes or strains. Interestingly, we have found that the presence of non-host D3 in strain FliC6 reduces the quality of χ phage binding and infection without abolishing it.

The structural factors within flagellin that determine χ susceptibility may be inclusionary or exclusionary in nature. Inclusionary would specify that a particular motif within NTD2, D3, or CTD2 is required for χ infection. Exclusionary would indicate that particular motifs block χ infection, and that a strain can be infected as long as its flagellin does not contain one of these exclusionary motifs. Van Asten et al. [75] reported that amino acid residues 258–348 of *S*. *enterica* serotype Enteritidis FliC form the epitope region determining the “g,m” flagellin antigen type. Meynell stated that χ infection in strains with flagellins of the g antigenic group is blocked [50], an observation that we have confirmed. It is therefore likely that certain residues within the “g,m” epitope region of FliC serve as an exclusionary factor preventing χ adsorption. The region identified by Van Asten et al. lies mostly within CTD2, with 32 residues (aa 327–348) extending into D3. This corroborates our initial hypothesis that the D2 domains are most important for the determination of χ susceptibility, while D3 plays a smaller role. Our detailed domain replacement strategy further supports this hypothesis. We have determined that a strain expressing ser. Enteritidis NTD2, D3, and CTD2 (FliC8) is fully resistant to χ and retains motility. This ser. Enteritidis substitution contains the entirety of the g,m epitope region. Additionally, the inverse mutant (FliC11) is susceptible to χ, as it contains the D2 and D3 domains from ser. Typhimurium with the “i” antigen epitope. This result suggests that the g,m epitope dictates χ susceptibility. We also discovered that substitution of 14028s FliC D3 with its ser. Enteritidis equivalent (FliC6) reduces the quality of phage binding and infectivity without abolishing it completely. Therefore, D3 seems less important than the D2 domains but may still be part of the exclusionary factor. This notion matches well with the fact that only 32 residues of the g.m epitope lie within D3. However, the non-motile phenotype of individual D2 domain mutants and the poorly defined span of most antigenic epitopes, including the “i” epitope of ser. Typhimurium FliC, are prohibitory to further investigations. We have also shown that many non-host serotypes do not produce g type flagellins, and that production of flagellin with a similar host-strain antigen does not guarantee a host phenotype. For instance, the non-host serotype Michigan does not produce g type flagellin and serotype Heidelberg is also a non-host despite producing flagellin of antigen type 1,2. Furthermore, phage χ also binds to the flagellar filament of its host *S*. *marcescens*, which has an antigenic region that is 136 amino acid residues shorter than the *S*. Typhimurium FliC equivalent [38]. In summary, we can conclude that factors within the D2 domains are essential, but we are unable to determine whether they are inclusionary or exclusionary. Furthermore, CTD2, as it compiles most of the epitope region, appears of greater importance for χ infection than NTD2. Finally, although a direct interaction of χ with the D3 domain is unlikely, exclusionary factors may exist that obstruct binding.

### Factors other than flagella influence host range

When ser. Enteritidis flagellin is expressed in a ser. Typhimurium Fla^-^ strain, motility is retained but infection is entirely abolished. However, the expression of ser. Typhimurium flagellin in an ser. Enteritidis Fla^-^ strain is sufficient to restore motility but the strain remains χ resistant (S1 Table). This implies that factors other than the flagellar filament structure are crucial in determining host susceptibility. Flagellin is by far the most structurally variable motility protein between *S*. *enterica* serotypes [37]. During χ translocation along the flagellum to the cell surface, it will first interact with the filament (FliC or FljB), then with the hook-filament junction (FlgLK), and lastly with the hook (FlgE). The primary sequences of serotypes Typhimurium and Enteritidis FlgK and FlgE are 100% identical, while FlgL differs by only one single residue [76,77]. This high sequence conservation implies that ser. Enteritidis resistance is likely the result of steps secondary to initial attachment and translocation along the flagellum. Recently, we identified that the multi-substrate efflux system AcrABZ-TolC is essential for χ infection of *S*. Typhimurium via a motility-unrelated mechanism [54]. We hypothesize that χ interacts with the AcrABZ-TolC complex at the cell surface and may use it as a channel to eject its DNA into the cytoplasm. RND-family inner membrane efflux pump AcrB, accessory protein AcrZ, and periplasmic adaptor AcrA are 100% identical, while the outer membrane channel TolC is 99% identical between the two serotypes. Further investigation is needed to determine whether the subtle differences between TolC of these two serotypes significantly affect susceptibility. We also discovered that the ribosome-associated molecular chaperone trigger factor is critical for χ phage infection of *S*. Typhimurium (over 95% reduction in EOP when absent), while deletion of the *tig* gene only caused a minor motility defect [54]. We hypothesize that trigger factor is involved in the folding of phage proteins, which would result in inefficient virion assembly in the absence of this chaperone. Since trigger factor proteins of both serotypes differ by only one amino acid residue, it is unlikely that this chaperone contributes to the functional differences in their χ resistance. Another significant difference between serotypes of *S*. *enterica* is the O antigen composition of lipopolysaccharide (LPS), which is a very common phage target. While it is possible that χ-resistant LPS mutants may have evaded our extensive deletion library screen analysis [54], as mutations in LPS are pleiotropic and often negatively affect motility [19,78,79], many factors generally disturb successful phage infection such as restriction/modification or CRISPR systems [80]. Overall, the χ phage infection process is very complex, and one or many other motility-unrelated distinctions between serotypes Enteritidis and Typhimurium likely play a role in the host phenotypes of these two serotypes.

### Analysis of mutant flagellin production

The immunoblot in Fig 5 provided evidence that all mutant strains produce their respective flagellin proteins, although it is unknown whether these are exported and assembled into a filament. The immunoblot analysis was successful despite several mutants having significantly altered epitope regions. However, it is worth noting that band intensities for flagellins with altered epitopes cannot be correlated directly with the amount of flagellin produced due to the antibody’s potentially reduced affinity for a particular mutant epitope. Interestingly, the non-motile mutants pFliC4, pFliC5, pFliC7, pFliC9, pFliC10, pFliC12, and pFliC13 produced bands not present in the Fla^-^ strain, indicating that FliC is stably produced, including highly truncated flagellin variants such as those lacking both D2 domains and additionally D3. We hypothesize that these significantly altered or truncated flagellins cannot be exported by the flagellar Type III export apparatus and/or are unable to form a filament.

While ser. Typhimurium FliC migrated according to its calculated molecular weight of 51 kDa, the 2-kDa larger ser. Enteritidis FliC migrated anomalously with an apparent molecular weight of 60 kDa. Interestingly, as revealed by the analysis of FliC domain variants, this phenomenon is caused specifically by the D3 domain of ser. Enteritidis FliC, as a FliC variant constructed on ser. Enteritidis FliC with D3 from ser. Typhimurium exhibits a migration behavior according to its calculated molecular weight (pFliC9). In contrast, pFliC10, which is ser. Enteritidis FliC containing ser. Typhimurium D2 domains, produces a FliC band that migrates at the anomalous apparent molecular weight of ser. Enteritidis FliC. The D3 domains in the two serotypes differ in length by only 10 residues, which would not cause such a significant apparent molecular weight shift. We can only speculate that this phenomenon is due to small variations in charge, as it is known that electrophoretic migration of methyl-accepting chemotaxis proteins is affected by neutralization of individual residues [81,82]. The ser. Enteritidis D3 domain possesses four additional positively charged and two additional negatively charged residues compared to its ser. Typhimurium equivalent [76,77].

The 34 kDa cross-reacting band is present in all sixteen samples including the Fla- strain. During antigen preparation, it is possible that another flagellar component had been co-purified with flagellin, allowing the resulting antiserum to recognize this protein. We hypothesize that this band corresponds to the hook-filament junction protein FlgL, which is 34.2 kDa in ser. Typhimurium, while the other proteins forming the hook and filament junction do not match this apparent molecular weight [25].

### Further studies, applications, and potential setbacks

Advanced knowledge about the interaction between χ and its host is necessary to eventually allow its inclusion in phage therapy cocktails. We have shown that the D2 domains of flagellin likely mediate the interaction with χ, possibly alongside D3. However, the domains or motifs within the χ receptor binding protein that are required for attachment to flagella are unknown. CHI_31, the putative tail fiber protein gene, could be mutagenized in a similar way as we have done for flagellin, to analyze the function of individual domains. It has not been shown experimentally that χ tail fiber consists of CHI_31, although the tail fiber protein has been shown to act as the RBP in the related phage YSD1 [60]. Furthermore, a high-resolution structure of the χ tail fiber is lacking, which would aid in the identification of potential targets for mutagenesis. An additional problem is the inherent difficulty in constructing targeted mutants of lytic bacteriophages. A CRISPR/Cas-based method is one of the currently utilized techniques to successfully mutagenize lytic phages [83]. Alternatively, a directed evolutionary approach may be effective and more straightforward. Such approaches have been applied to isolate host range mutants of bacteriophages [84]. Alternatively, chemical mutagens like ethyl methanesulfonate or 5-bromouracil could be used to mutate phage DNA [85,86]. UV mutagenesis could also be attempted, as this has been applied to *E*. *coli* phage λ [87].

In this study, we have characterized part of the complex interaction between flagellotropic phage χ and *S*. *enterica* flagellin. We have demonstrated that alteration of certain FliC domains is sufficient to fully block χ infection. Our data, combined with previous knowledge about the flagellin g antigen epitope region, lead to our hypothesis that CTD2 is the most crucial domain for χ binding. Domains D3 and NTD2 are likely also significant but less important factors. Since g antigen flagellins block χ infection [50], we believe that exclusionary factors exist within CTD2 or the C-terminal end of D3, which are part of the g epitope. Our data support this hypothesis, as exchange of both D2 domains and the D3 domain leads to a complete switch of the χ susceptibility phenotype, but alteration of solely D3 has a comparatively minor effect. However, we realize that there are numerous serotypes expressing non-g flagellins that are also entirely χ resistant (S1 Table), so there are clearly more factors involved in determining host range. Multiple studies have shown that mutagenesis can be used to alter a phage’s host range [84,88]. This has clear potential in clinical applications. We now know that subtle changes in flagellin structure can significantly alter the ability of χ to bind flagella and subsequently kill its host. Findings of this study will move research in the direction of genetically modified phage treatments in clinical applications, a promising technique that has only recently come to fruition.

## Materials and methods

### Strains and plasmids

Strains and plasmids used in this study are listed in Table 3.

**Table 3:** Plasmids, bacterial strains, and bacteriophage used in this study. Fig 1 provides further detail on flagellin domain mutants. *S*. *enterica* serotypes tested for qualitative χ susceptibility are not included in this table and are listed comprehensively in S1 Table.

Strain/plasmid	Parent strain/plasmid	Genotype/ Relevant characteristics	Source
pTrc99a	pBR322	P_lac_; MCS; pBR322 ori; amp^r^	Howard C. Berg
pBS1316	pTRC99a	P_fliC(14028s)_; MCS; pBR322 ori; amp^r^	This study
pFliC1	pBS1316	See Fig 1	This study
pFliC2	pBS1316	See Fig 1	This study
pFliC3	pBS1316	See Fig 1	This study
pFliC4	pBS1316	See Fig 1	This study
pFliC5	pBS1316	See Fig 1	This study
pFliC6	pBS1316	See Fig 1	This study
pFliC7	pBS1316	See Fig 1	This study
pFliC8	pBS1316	See Fig 1	This study
pFliC9	pBS1316	See Fig 1	This study
pFliC10	pBS1316	See Fig 1	This study
pFliC11	pBS1316	See Fig 1	This study
pFliC12	pBS1316	See Fig 1	This study
pFliC13	pBS1316	See Fig 1	This study
pWRG730	pSIM5	P_L_ *redαβγ*; P_tet_ I-SceI; ori SC101_TS_; chl^r^	Roman Gerlach
pWRG717	pBluescript II	kan/I-SceI cassette template plasmid; kan^r^	Roman Gerlach
14028s	*Salmonella enterica* subsp. *enterica* serotype Typhimurium 14028s	Wild type	Michael McClelland
14028s Δ*fliB*	14028s	Δ*fliB*	This study
14028s Δ*fliB* pWRG730	14028s	Δ*fliB*; pWRG730; chl^r^	This study
14028s fliC_ON_ Δ*fliB*	14028s	Δ*fliB*; Δ*fljBAhin*::*tetRA*; tet^r^	This study
14028s fliC_ON_ Δ*fliB* pWRG730	14028s	Δ*fliB*; Δ*fljBAhin*::*tetRA*; pWRG730; chl^r^ tet^r^	This study
FliC1	14028s fliC_ON_ Δ*fliB*	See Fig 1	This study
FliC2	14028s fliC_ON_ Δ*fliB*	See Fig 1	This study
FliC3	14028s fliC_ON_ Δ*fliB*	See Fig 1	This study
FliC6	14028s fliC_ON_ Δ*fliB*	See Fig 1	This study
FliC8	14028s fliC_ON_ Δ*fliB*	See Fig 1	This study
FliC11	14028s fliC_ON_ Δ*fliB*	See Fig 1	This study
14028s Fla^-^	14028s	Δ*fliC*::*kan* Δ*fljBAhin*::*tetRA*; tet^r^; kan^r^	[54]
P125109	*Salmonella enterica* subsp. *enterica* serotype Enteritidis	Wild type	Michael McClelland
TH2788	*Salmonella* Typhimurium LT2	*fliY*::Tn10dTc; tet^r^	Kelly T. Hughes
DH5α	*Escherichia coli* K12	F^−^*endA1 glnV44 thi-1 recA1 relA1 gyrA96 deoR nupG purB20* φ80d*lacZ*ΔM15 Δ(*lacZYA-argF*)U169, *hsdR17*(*r*_*K*_^−^*m*_*K*_^+^), λ^–^	Howard C. Berg
Bacteriophage χ	-	Wild type	Saeed Tavazoie

### Construction of cloning vector pBS1316

Flagellin expression vector pBS1316 was constructed using Gibson assembly [89]. Oligonucleotides (Invitrogen) were designed to amplify plasmid pTrc99a, retaining its multiple cloning site. Additional primers were designed to amplify the 146 bases immediately upstream of the ser. Typhimurium *fliC* ATG start codon, ensuring coverage of the entirety of the *fliC* promoter and known regulatory elements [28,63]. Primers additionally included 40-bp of flanking homology to the ends of the linearized pTrc99a. These purified PCR products were assembled using NEB 2x Gibson Assembly Master Mix and *E*. *coli* DH5α was subsequently transformed with the assembled reaction mixture and plated on lysogeny broth (LB) plates [90] (10 g/l tryptone, 5 g/l yeast extract, 5 g/l NaCl, 15 g/l agar) with 100 μg/ml ampicillin (LB amp). The plasmid construct was verified by Sanger sequencing.

### Trans-complementation of flagellin in 14028s

The *fliC* mutant inserts were generated by PCR. Domains from either *S*. Typhimurium 14028s or *S*. Enteritidis P125109 were amplified and fused together by overlap extension PCR using outer primers with BamHI and SalI restriction enzyme sites. Some more complex mutants required multiple overlap extension steps. Plasmid pBS1316 and each completed insert were digested with these enzymes, purified via 1% TAE-agarose gel electrophoresis and subsequent kit purification (Thermo Scientific GeneJet), and ligated using T4 DNA ligase (New England Biolabs). *E*. *coli* DH5α was transformed with the ligation mixtures and constructs were verified by Sanger sequencing. Electrocompetent *S*. Typhimurium 14028s Fla^-^ cells were prepared by first growing bacteria at 37°C until an OD_600_ of 0.3–0.5 was reached. Cells were then chilled on ice for 15 minutes and centrifuged at 8,000 x g for 10 minutes at 4°C. The supernatant was discarded and cells were washed twice with 1/2 volume of ice-cold 10% glycerol, then resuspended in 1/100^th^ volume of 10% glycerol. Electrotransformation was performed by first adding 100 ng of pBS1316 construct plasmid DNA to a 50 μl aliquot of electrocompetent cells. This was transferred to a 0.2 cm electroporation cuvette (Bio-Rad) and electrotransformed via a 5 ms exponential-decay pulse at 2.5 kV, 25 μF, 200 Ω. One ml of chilled SOC medium (20 g/l tryptone, 5 g/l yeast extract, 10 mM NaCl, 2.5 mM KCl, 10 mM MgCl_2_, 10 mM MgSO_4_, 20 mM glucose) was immediately added and the cell suspension was plated on LB amp after a 1-hour recovery at 37°C. Motility of these Fla^-^ pFliC mutants was qualitatively determined via microscopy.

### Mutant construction

Trans-complementation was used to decide which mutations to construct chromosomally. A corresponding chromosomal mutant was not constructed for plasmid mutants that were entirely non-motile. Trans-complementation mutants found to be motile had their mutant alleles amplified from the plasmid and inserted into the chromosome using the lambda-red recombineering system and I-SceI nuclease counterselection essentially as described previously [91]. Fresh electrocompetent cells were prepared for each transformation. Plasmid pWRG730 (P_L_ λred; P_tet_ I-SceI; ori SC101_TS_) [91] was introduced into ser. Typhimurium 14028s by electroporation and cells were recovered in 1 ml terrific broth (20 g/l tryptone, 24 g/l yeast extract, 0.4% v/v glycerol, 17 mM KH_2_PO_4_, 72 mM K_2_HPO_4_) for 60 minutes at 30°C. The cell suspension was spread plated on LB with 10 μg/ml chloramphenicol (LB chl_10_) and incubated overnight at 30°C.

For *fliC* allelic exchange, oligonucleotides were designed with 40-bp flanking homology to sequences within *fliC* and 20-bp of homology to template plasmid pWRG717 [91]. PCR was used to amplify the kan/I-SceI cassette from pWRG717 using Phusion DNA polymerase (New England Biolabs). This cassette contains the aminoglycoside phosphotransferase gene conferring kanamycin resistance, and the I-SceI nuclease recognition site TAGGGATAACAGGGTAAT. Strain 14028s with pWRG730 was grown at 30°C until an OD_600_ of 0.5 was reached. This culture was then transferred to a 42°C shaking water bath incubator for 15 minutes to induce expression of lambda-red genes under the λ P_L_ promoter, followed by immediately transferring the culture to ice. Cells were made electrocompetent as described above, and the kan/I-SceI cassette with 40 bp *fliC* flanking homology was introduced by electroporation followed by recovery in terrific broth at 30°C for 1 hour and plating on LB with 50 μg/ml kanamycin and 10 μg/ml chloramphenicol (LB kan/chl_10_) at 30°C to select for insertion mutants and retain plasmid pWRG730. Kan/chl-resistant colonies were isolated and *fliC*::kan/I-SceI insertion in one colony was verified by colony PCR. Mutant *fliC* DNA was amplified from the respective pBS1316 template plasmid and electrocompetent 14028s *fliC*::kan/I-SceI strain was transformed with the mutant *fliC* PCR product and recovered at 30°C in terrific broth for 1 hour. Serial 1:10 dilutions were prepared and 10^−1^ through 10^−4^ were spread plated on LB with 30 μg/ml chloramphenicol and 0.5 μg/ml anhydrotetracycline (aTc) (LB chl_30_/aTc) at 30°C. After overnight incubation, large colonies were streaked for isolation on LB chl_30_/aTc and subsequently streaked on LB kan/chl_10_ to confirm loss of kanamycin resistance. Kan^s^ colony *fliC* genotypes were then verified by colony PCR and Sanger sequencing. To eliminate the variable of flagellin lysine methylation, the flagellin methylase gene *fliB* was deleted scarlessly in the same manner. The kan/I-SceI cassette was inserted into the *fliB* gene and subsequently crossed out with the second step cassette consisting of a fusion of 500 bp immediately up and downstream of the *fliB* gene followed by selection on LB chl_30_/aTc plates. The absence of the *fliB* gene was confirmed by colony PCR and Sanger sequencing. To eliminate flagellin phase variation, the genes *fljB* and *fljA* were inactivated by insertion of a single *tetRA* cassette amplified from TH2788 (*S*. Typhimurium LT2 *fliY*::Tn10dTc) with flanking homology to the beginning of *fljB* and the end of *fljA* and subsequent selection on LB with 10 μg/ml tetracycline (LB tet). After mutagenesis was complete, pWRG730 was cured from the strain by overnight incubation at 42°C on LB tet, followed by confirmation of the chl^s^/kan^s^/tet^r^ phenotype.

### Verification and quantification of motility

Motility of cells grown to an OD_600_ of 2.0 was verified using phase contrast microscopy. Quantitative motility assays were conducted on soft agar swim plates containing MSB swim medium (1% tryptone, 0.5% yeast extract, 0.3% agar, and 2 mM each of MgSO_4_ and CaCl_2_). Each *S*. *enterica* strain was inoculated into 10 ml of LB and placed into a 37°C shaking incubator at 220 RPM overnight. A 2.5 μl drop of the stationary phase bacterial culture was pipetted onto the center of a soft agar swim plate. This was repeated in technical triplicate for each of three biological replicates. The plates were incubated at 37°C and measured at two different time points, after eight and 16 hours.

### Spot assays for qualitative determination of phage susceptibility

*Salmonella* serotypes of interest were grown in LB medium until an OD_600_ of 2.0 was reached. Cell motility was verified via phase contrast microscopy. Non-motile strains were not tested; all non-motile bacteria are completely χ resistant because rotating flagella must be present for infection to proceed [49]. Next, 100 μl of motile cell suspension was mixed with 4 ml of molten 0.5% agar LB and immediately poured onto a 1.5% agar LB plate. Agar was allowed to solidify and serial 1:10 dilutions of χ phage were prepared in 0.85% NaCl. Ten microliters of each dilution were spotted onto the soft agar lawn and plates were left on the benchtop until fully dry, then flipped, and incubated at 37°C overnight. Zones of clearing indicated phage-mediated cell lysis and thus susceptibility.

### Plaque assays for calculation of EOP

Plaque assays were performed using standard procedures. Briefly, cells were grown to an OD_600_ of 2.0 and verified motile via phase contrast microscopy as described above. Bacteriophage χ was diluted 1:10 serially in 0.85% NaCl. One hundred microliters of motile cells in LB were mixed with 100 μl of phage dilutions and allowed to incubate for six minutes to allow binding. Next, 4 ml of molten 0.5% agar LB was mixed with the cell-phage mixture, which was then poured onto 1.5% agar LB plates and allowed to solidify. Plaques were counted after overnight incubation at 37°C. EOP was calculated using the formula EOP = (pfu/ml mutant)/(pfu/ml WT)*100%.

### Assay of phage adsorption

To determine adsorption percentage, the *Salmonella* strain of interest was grown in triplicate in 10 ml LB with 220 RPM shaking at 37°C to an OD_600_ of 2.0. Culture motility was verified by phase contrast microscopy. A 10 ml LB control with no cells was also prepared. Next, 10^6^ pfu of χ phage was added to each culture. The cultures were returned to shaking for ten minutes to allow binding to occur. One ml of each culture was centrifuged at 15,000 x g for 3 minutes at 4°C. Supernatants were removed without disturbing the cell pellets and serial 1:10 dilutions were immediately prepared. Phages present in supernatants were titered via plaque assay, using *S*. Typhimurium 14028s as indicator strain. Adsorption percentage was calculated using the formula: (1 –(pfu supernatant with cells/pfu control) * 100%).

### Gel electrophoresis and immunoblot analysis for determination of flagellin production

Immunoblot analysis was employed to determine whether flagellin is produced by a particular pFliC mutant. In addition, whole cell lysates were also visualized on SDS-PAGE gels stained with Coomassie brilliant blue R250 (S2 Fig). First, pFliC mutant strains were grown to an OD_600_ of 2.0. Motility was verified by phase contrast microscopy for the strains that were known to be motile. Next, 15 μl of each culture was mixed with 15 μl of 2x Laemmli loading buffer (65.8 mM Tris pH 6.8, 2.1% SDS, 26.3% glycerol, 0.01% bromophenol blue, 5% β-mercaptoethanol) and boiled for 10 minutes, before loading on a Criterion TGX 10% polyacrylamide gel (Bio-Rad). Precision Plus Protein dual-color molecular weight marker (Bio-Rad) was loaded to determine protein size. After gel electrophoresis, proteins were transferred to a 0.45 μm nitrocellulose membrane (Cytiva). The membrane was blocked overnight by incubating in a solution of 5% nonfat dry milk in phosphate-buffered saline with Tween 20 (PBS-T; 80 mM Na_2_HPO_4_, 20 mM NaH_2_PO_4_, 100mM NaCl, 0.1% v/v Tween 20) on a rocking platform. BD Difco *Salmonella* H Antiserum a-z was added to freshly-prepared blocking solution at a ratio of 1:5,000 and this mixture was incubated with the membrane for two hours under gentle agitation. The membrane was washed four times with PBS-T and incubated with freshly-prepared blocking solution containing 1:2,500 secondary StarBright Blue 700 Goat Anti-Rabbit IgG (Bio-Rad) for one hour, agitating gently. After four washes with PBS-T, the blot was imaged with a Bio-Rad Chemidoc instrument. Controls consisted of ser. Typhimurium 14028s FliC_ON_/Δ*fliB* (FliC1), ser. Enteritidis P125109 WT, and ser. Typhimurium 14028s Fla^-^.

## Supporting information

S1 Table Comprehensive list of *Salmonella enterica* serotypes tested for χ phage susceptibility by spot assay, and their typical phase 1 (FliC), phase 2 (FljB), and phase 3 (FlpA) flagellin (H) antigenic formulae, where applicable, as described by literature sources. The symbol (-) indicates the absence of this specific flagellin gene. Formation of a lysis zone when χ was spotted on a bacterial lawn indicated a susceptible phenotype. No lysis zone formation was interpreted as χ resistance. All are serotypes of *Salmonella enterica*; serotypes that are given names are part of subsp. enterica, while serotypes given only antigenic designations are other S. enterica subspecies. Non-motile serotypes are not included. Also included are a ser. Enteritidis fliC deletion mutant complemented by pFliC1, a ser. Typhimurium FliC monophasic strain, and a ser. Typhimurium FljB monophasic strain.
(DOCX)

S2 Table Comprehensive raw data for soft agar swim plates, efficiency of plating assay, and phage adsorption assay experiments. (XLSX)

S1 Fig Amino acid sequence alignment of *Salmonella enterica* flagellin proteins with domains highlighted. Top: ser. Typhimurium flagellin FliC. Bottom: ser. Enteritidis flagellin FliC. D0 domains are highlighted in blue, D1 domains are highlighted in red, D2 domains are highlighted in yellow, and D3 is highlighted in green. Residue numbers, percent identity, and percent similarity are given in Table 2. Sequences were aligned using EMBOSS Needleman-Wunsch algorithm.
(TIF)

S2 Fig Coomassie blue-stained denaturing 4–20% polyacrylamide gel of whole cell lysates from pFliC mutants grown to an OD_600_ of 2.0. Ser. Typhimurium 14028s FliC_ON_ Δ*fliB* (chromosomal mutant FliC1) is denoted as “WT.” Wild type ser. Enteritidis P125109 is denoted as “Ent.” Ser. Typhimurium 14028s Δ*fliC*Δ*fljBA* is denoted as “Fla^-^.” Numbers 1 through 13 refer to the corresponding pFliC plasmid mutants, as described in Fig 1. Bands likely corresponding to flagellin are labelled with a red dot on the rightmost end of the band. Lanes with no marked band do not exhibit a distinct flagellin band or are ambiguous.
(TIF)
