# Supplementary material for: Phages on filaments: A genetic screen elucidates the complex interactions between Salmonella enterica flagellin and bacteriophage Chi
Source: PLoS Pathog. 2023 Aug 3;19(8):e1011537. doi: 10.1371/journal.ppat.1011537 (PMC10399903; doi:10.1371/journal.ppat.1011537)
Supplement: S1 Table — The symbol (-) indicates the absence of this specific flagellin gene. Formation of a lysis zone when χ was spotted on a bacterial lawn indicated a susceptible phenotype. No lysis zone formation was interpreted as χ resistance. All are serotypes of Salmonella enterica; serotypes that are given names are part of subsp. enterica, while serotypes given only antigenic designations are other S. enterica subspecies. Non-motile serotypes are not included. Also included are a ser. Enteritidis fliC deletion mutant complemented by pFliC1, a ser. Typhimurium FliC monophasic strain, and a ser. Typhimurium FljB monophasic strain. (DOCX) [file ppat.1011537.s001.docx]

S1 Table. Comprehensive list of Salmonella enterica serotypes tested for χ phage susceptibility by spot assay, and their typical phase 1 (FliC), phase 2 (FljB), and phase 3 (FlpA) flagellin (H) antigenic formulae, where applicable, as described by literature sources. The symbol (-) indicates the absence of this specific flagellin gene. Formation of a lysis zone when χ was spotted on a bacterial lawn indicated a susceptible phenotype. No lysis zone formation was interpreted as χ resistance. All are serotypes of Salmonella enterica; serotypes that are given names are part of subsp. enterica, while serotypes given only antigenic designations are other S. enterica subspecies. Non-motile serotypes are not included. Also included are a ser. Enteritidis fliC deletion mutant complemented by pFliC1, a ser. Typhimurium FliC monophasic strain, and a ser. Typhimurium FljB monophasic strain.

| **Serotype/antigenic designation** | **Phase 1 H antigen** | **Phase 2 H antigen** | **Phase 3 H antigen** | **Result** |
| --- | --- | --- | --- | --- |
| Abortusovis | c | 1,6 | - | Resistant |
| Agona | f,g,s | 1,2 | z_27_,z_45_ | Resistant |
| Anatum | e,h | 1,6 | z_64_ | Resistant |
| Bovismorbificans | r,i | 1,5 | R1 | Susceptible |
| Choleraesuis | c | 1,5 | - | Susceptible |
| Enteritidis | g,m | - | - | Resistant |
| Enteritidis Δ*fliC* pFliC1 | i | - | - | Resistant |
| Hadar | z_10_ | e,n,x | - | Susceptible |
| Hartford | y | e,n,x | z_67_ | Resistant |
| Heidelberg | r | 1,2 | - | Resistant |
| Infantis | r | 1,5 | R1,z_37_,z_45_,z_49_ | Susceptible |
| Java | b | 1,2 | z_5_,z_33_ | Susceptible |
| Javiana | l,z_28_ | 1,5 | R1 | Susceptible |
| Kottbus | e,h | 1,5 | - | Resistant |
| Meleagridis | e,h | l,w | - | Resistant |
| Miami | a | 1,5 | - | Susceptible |
| Michigan | l,v | 1,5 | - | Resistant |
| Montevideo | g,m,p,s | 1,2,7 | - | Resistant |
| Muenchen | d | 1,2 | z_67_ | Resistant |
| Newport | e,h | 1,2 | z_67_,z_78_ | Susceptible |
| Oranienburg | m,t | z57 | - | Resistant |
| Paratyphi B | b | 1,2 | z_5_,z_33_ | Susceptible |
| Poona | z | 1,6 | z_44_,z_59_ | Susceptible |
| Rubislaw | r | e,n,x | - | Susceptible |
| Saintpaul | e,h | 1,2 | - | Resistant |
| Schwarzengrund | d | 1,7 | - | Susceptible |
| Seftenberg | g,s,t | - | z_27_,z_34_,z_37_,z_43_, z_45_,z_46_,z_82_ | Resistant |
| Stanley | d | 1,2 | - | Susceptible |
| Thompson | k | 1,5 | R1 | Resistant |
| Typhimurium | i | 1,2 | - | Susceptible |
| Typhimurium FliC-ON | i | - | - | Susceptible |
| Typhimurium FljB-ON | - | 1,2 | - | Susceptible |
| Weltevreden | r | z6 | - | Susceptible |
| 3a:41:z_4_,z_23_:- | z_4_,z_23_ | - | - | Susceptible |
| 3a:48:z_4_,z_23_:- | z_4_,z_23_ | - | - | Susceptible |
| 3b:61:1,v:1,5 | 1,v | 1,5 | - | Susceptible |
| 6:45:a:e,n,x | a | e,n,x | - | Susceptible |
| 6:11:b:e,n,x | b | e,n,x | - | Resistant |
| 2:47:b:1,5 | b | 1,5 | - | Resistant |
| 2:58:d:_Z6_ | d | z6 | - | Resistant |
