# Supplementary figures and images for: Phages on filaments: A genetic screen elucidates the complex interactions between Salmonella enterica flagellin and bacteriophage Chi
Source: PLoS Pathog. 2023 Aug 3;19(8):e1011537. doi: 10.1371/journal.ppat.1011537 (PMC10399903; doi:10.1371/journal.ppat.1011537)

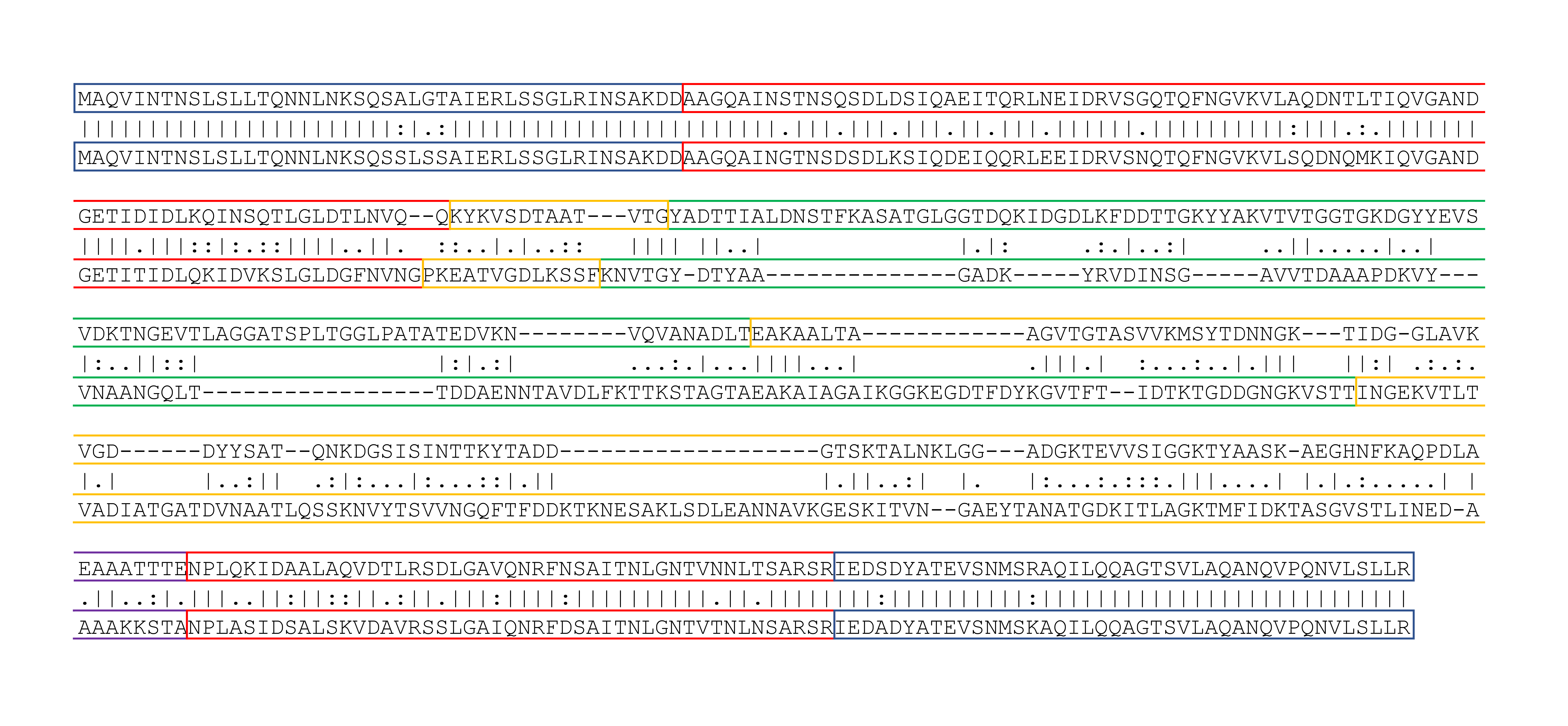

Supplement: S1 Fig — Top: ser. Typhimurium flagellin FliC. Bottom: ser. Enteritidis flagellin FliC. D0 domains are highlighted in blue, D1 domains are highlighted in red, D2 domains are highlighted in yellow, and D3 is highlighted in green. Residue numbers, percent identity, and percent similarity are given in Table 2. Sequences were aligned using EMBOSS Needleman-Wunsch algorithm. (TIF) [file ppat.1011537.s003.tif]

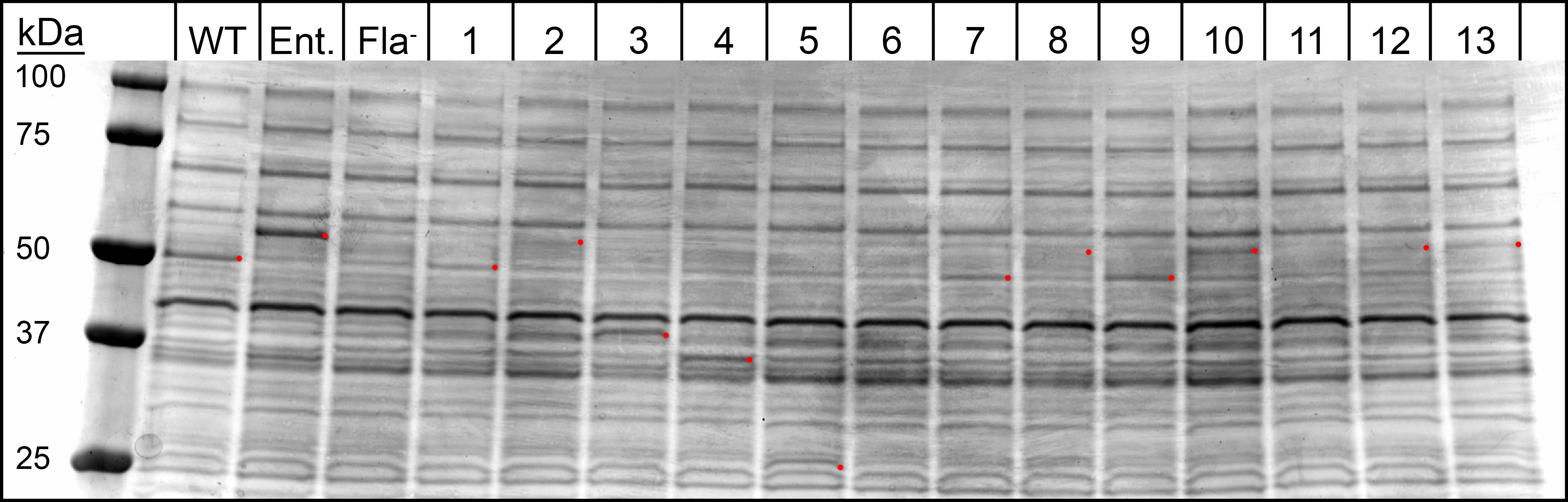

Supplement: S2 Fig — Ser. Typhimurium 14028s FliCON ΔfliB (chromosomal mutant FliC1) is denoted as “WT.” Wild type ser. Enteritidis P125109 is denoted as “Ent.” Ser. Typhimurium 14028s ΔfliCΔfljBA is denoted as “Fla-.” Numbers 1 through 13 refer to the corresponding pFliC plasmid mutants, as described in Fig 1. Bands likely corresponding to flagellin are labelled with a red dot on the rightmost end of the band. Lanes with no marked band do not exhibit a distinct flagellin band or are ambiguous. (TIF) [file ppat.1011537.s004.tif]
